# Supplementary material for: Genomic resources for the scuttle fly Megaselia abdita: a model organism for comparative developmental studies in flies
Source: Development. 2025 Nov 24;152(22):dev204732. doi: 10.1242/dev.204732 (PMC12687330; doi:10.1242/dev.204732)
Supplement: Supplementary information [file develop-152-204732-s1.pdf]

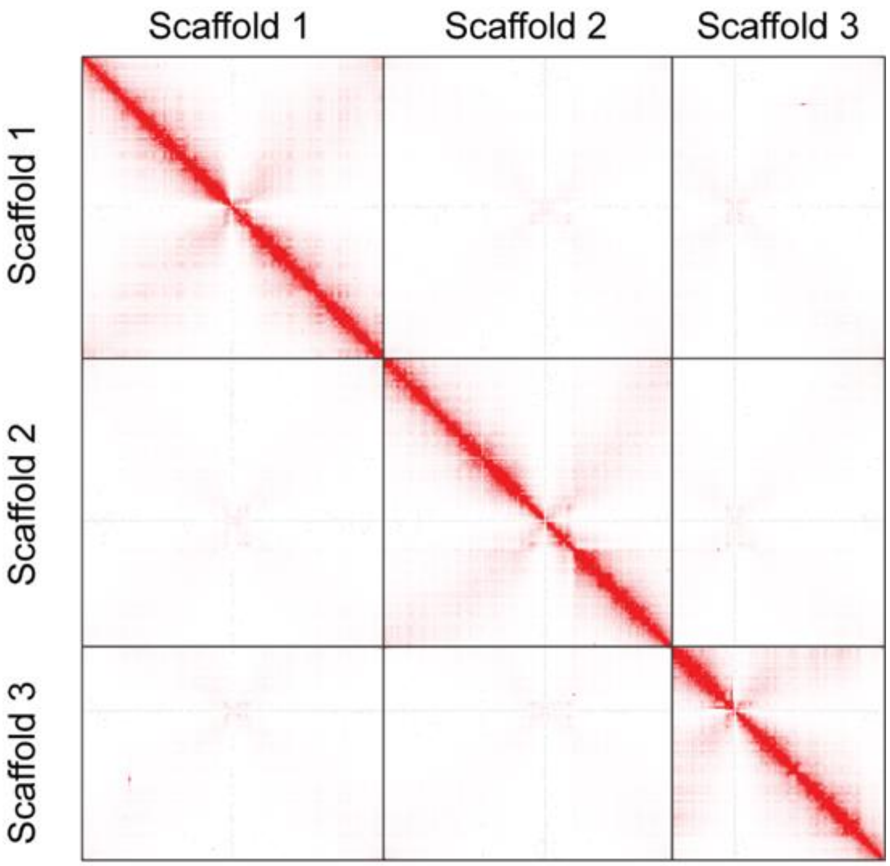

Fig. S1. Hi-C contact map showing spatial proximity between scaffolds in the genome assembly. The intensity of red indicates the frequency of interactions between genomic regions, with darker red representing higher contact frequencies. Scaffolds are arranged along the axes, and strong diagonal signals indicate intra-scaffold interactions.

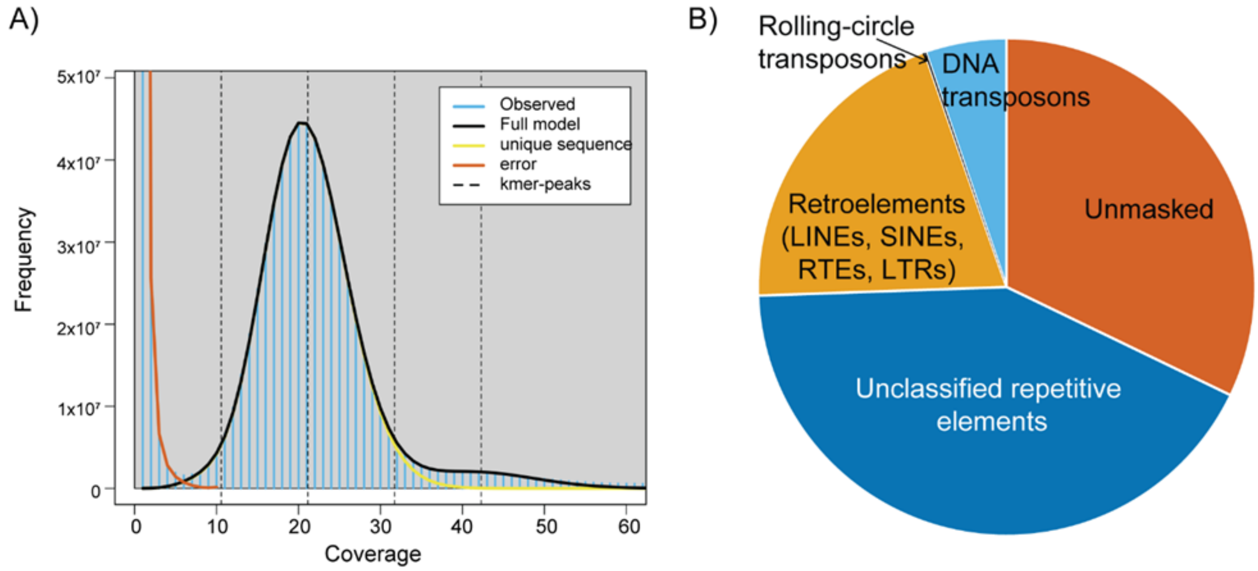

Fig. S2. *M. abdita* genome characteristics: k-mer distribution and repetitive element composition.

A) 21-mer frequency distribution for *M. abdita* PacBio reads. The observed k-mer coverage (blue) is modeled by GenomeScope (black line), which includes contributions from unique sequences (yellow) and sequencing errors (orange). In this context, 'coverage' on the x-axis refers to the number of times a k-mer is observed in the sequencing reads and 'frequency' on the y-axis to the number of unique k-mers with that number of observations. In our data, we see ~4.5×10^7 unique 21-mers (frequency) that were observed ~20 times (coverage).

B) Proportion of the *M. abdita* genome corresponding to repetitive elements. Retrotransposons (e.g., LINEs, SINEs, RTEs, LTRs), DNA transposons, rolling-circle transposons, unclassified repetitive elements, and unmasked regions are also included.

Development • Supplementary information

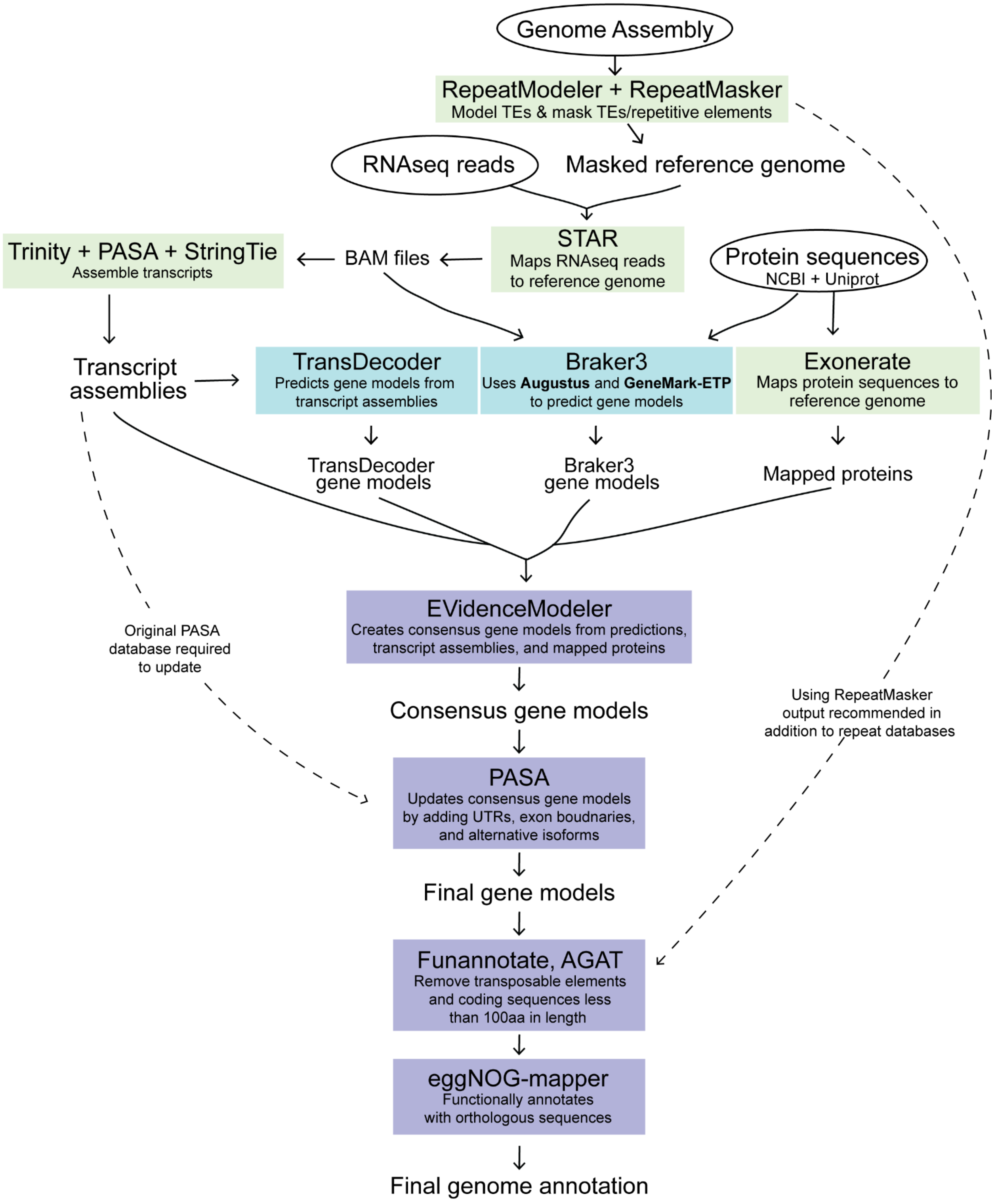

Development • Supplementary information

Fig. S3. Genome annotation pipeline. The starting input files are shown in black ovals and include the genome assembly along with two lines of evidence: RNA-Seq reads (fastq format) and protein sequences obtained from NCBI and UniProt. Software tools are represented in colored boxes: green indicates mapping software, blue indicates gene model generation software, and purple indicates post-gene model processing and functional annotation tools. Arrows pointing from a software box to unboxed text represent the output files generated by the software. Arrows leading from unboxed text to a software box indicate input files used by the software.

Development • Supplementary information

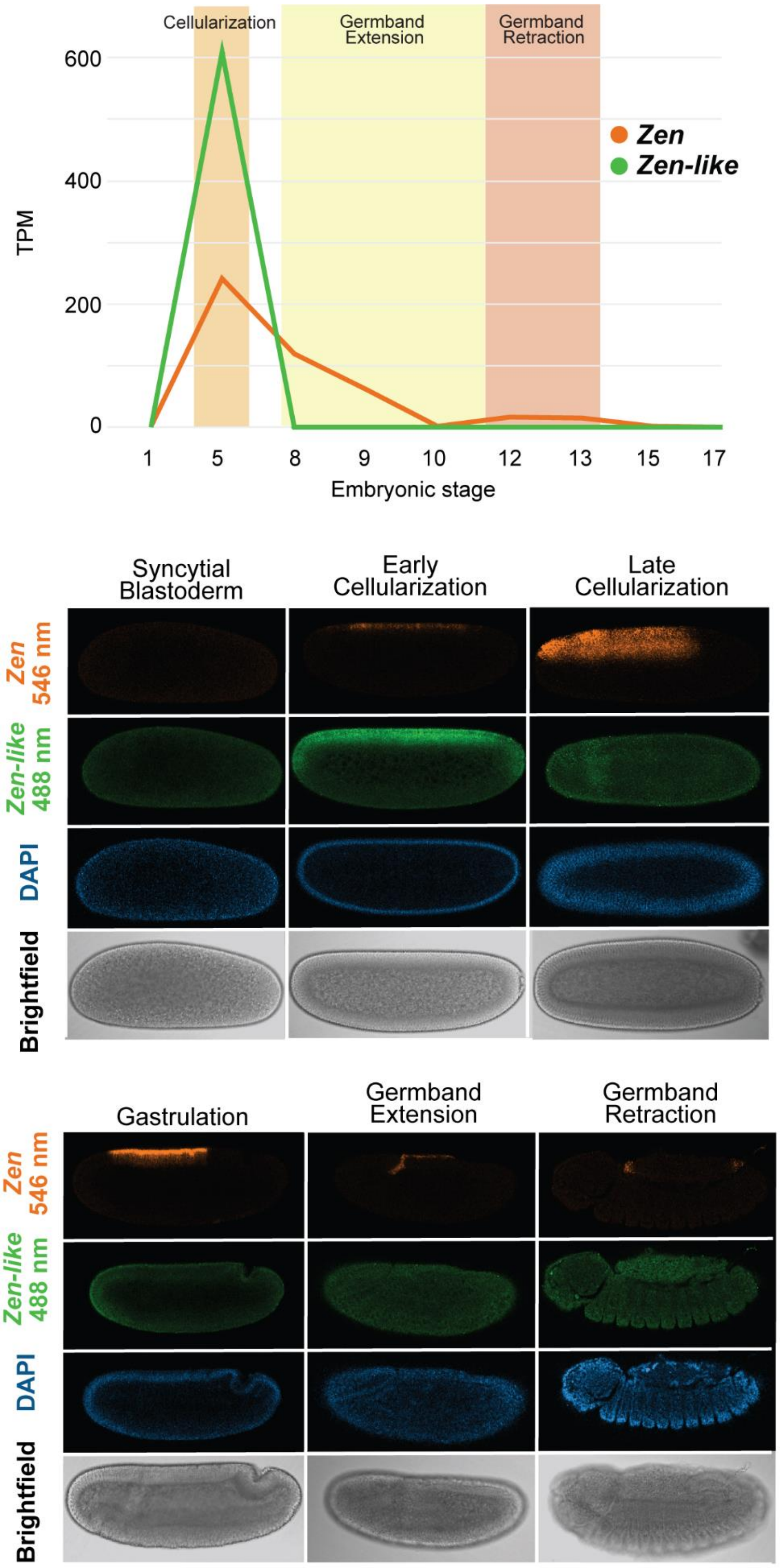

Cellularization
Germband Extension
Germband Retraction
600
400
200
0
TPM
Zen
Zen-like
1
5
8
9
10
12
13
15
17
Embryonic stage
Syncytial Blastoderm
Early Cellularization
Late Cellularization
Zen 546 nm
Zen-like 488 nm
DAPI
Brightfield
Gastrulation
Germband Extension
Germband Retraction
Zen 546 nm
Zen-like 488 nm
DAPI
Brightfield

Fig. S4. A) Expression of *Zen* (orange) and *Zen-like* (green) genes in *M. abdita* across embryonic developmental stages. Expression is shown in transcripts per million (TPM). B) Fluorescent *in situ* hybridization chain reaction (HCR) of *zen* (orange) and *zen-like* (green) transcripts in six developmental stages of *M. abdita* embryos. Shown are representative single z-planes acquired at 20× magnification; anterior is left, dorsal up. Stages correspond to syncytial blastoderm, stage 5 (early and late cellularization), stage 7 (gastrulation), stage 9 (germband extension), and stage 13 (germband retraction). DAPI (blue) and brightfield images are included to aid staging and orientation. *Zen* is expressed during cellularization and gastrulation, whereas *zen-like* is expressed only during early cellularization. Anterior is left and dorsal up.

Development • Supplementary information

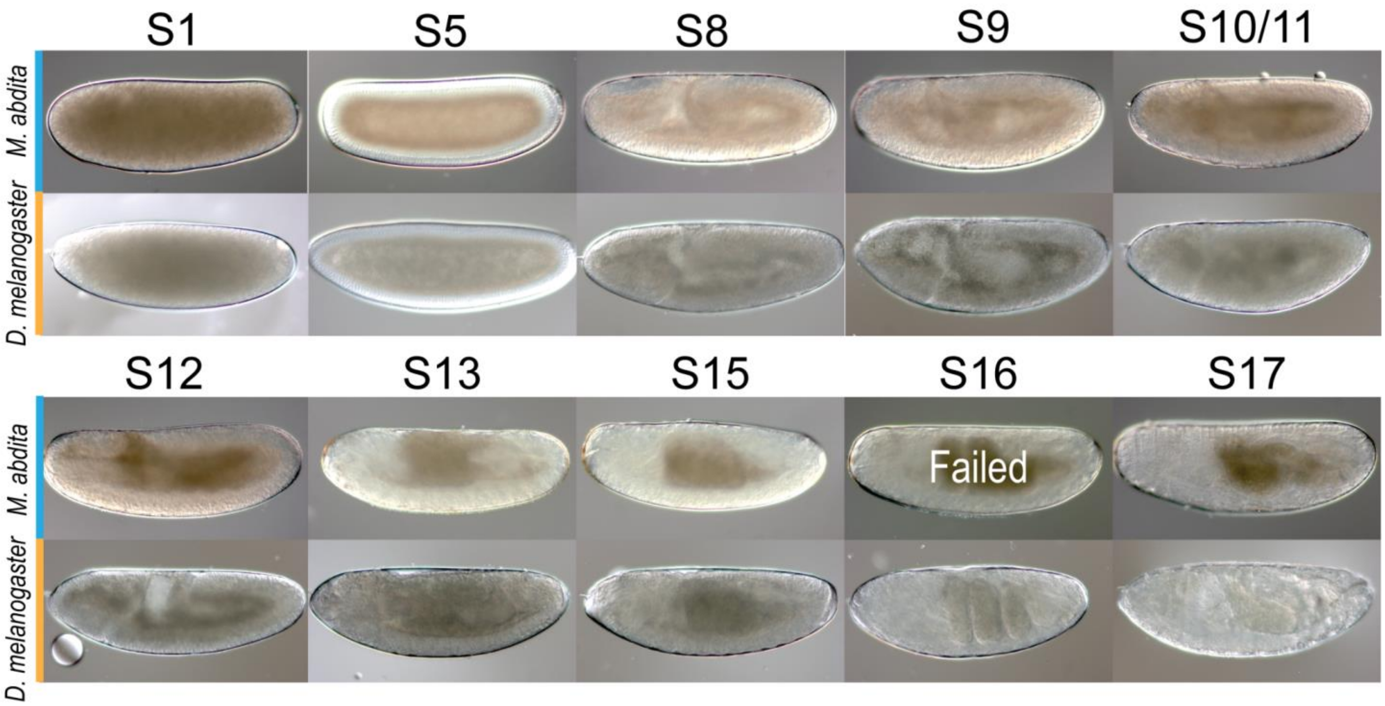

Fig. S5. Images of the embryos used for individual RNA sequencing, spanning developmental stages 1 to 17. *M. abdita* embryos are shown in the top row (blue bar) and *D. melanogaster* embryos in the bottom row (yellow bar). Each stage is indicated above the corresponding images. Note that for stage 16 of *M. abdita*, sequencing failed. Anterior is left and dorsal up.

Development • Supplementary information

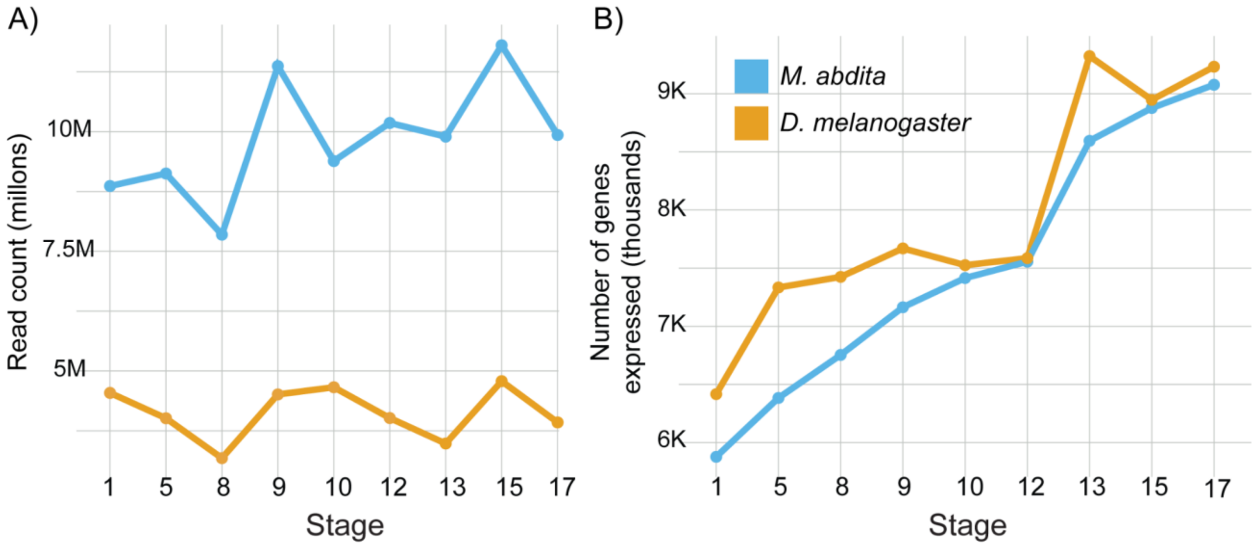

Fig. S6. RNA-seq read counts and gene expression across embryonic stages in *D. melanogaster* and *M. abdita.*

A) Comparison of raw RNA-seq read counts for single embryos of *D. melanogaster* (yellow) and *M. abdita* (blue) across embryonic developmental stages.

B) Comparison of the number of genes expressed in single embryos of *D. melanogaster* (yellow) and *M. abdita* (blue) across embryonic developmental stages.

Development • Supplementary information

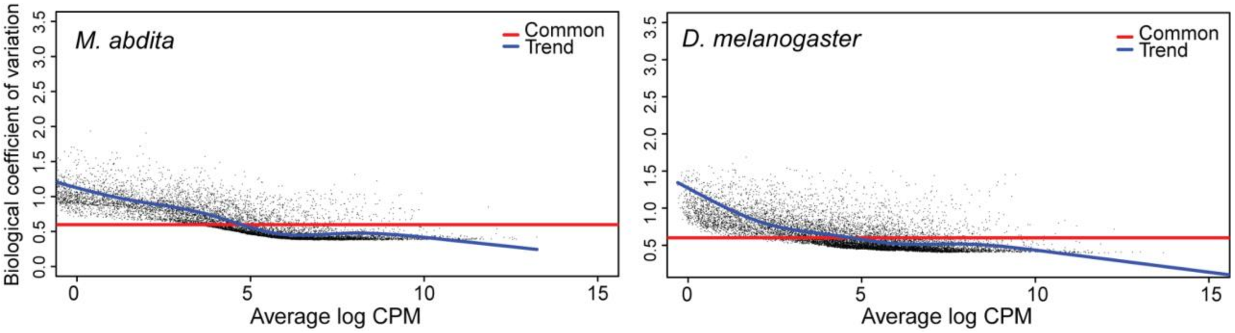

Fig. S7. Biological coefficient of variation (BCV) plots for *M. abdita* (left) and *D. melanogaster* (right) RNA-seq data. Each black dot represents a gene, with the BCV plotted against the average log counts per million (log_2CPM). The red line indicates the global common dispersion (BCV^2 is equal to dispersion), while the blue line shows the trend of gene-specific dispersions as a function of expression level.

Development • Supplementary information

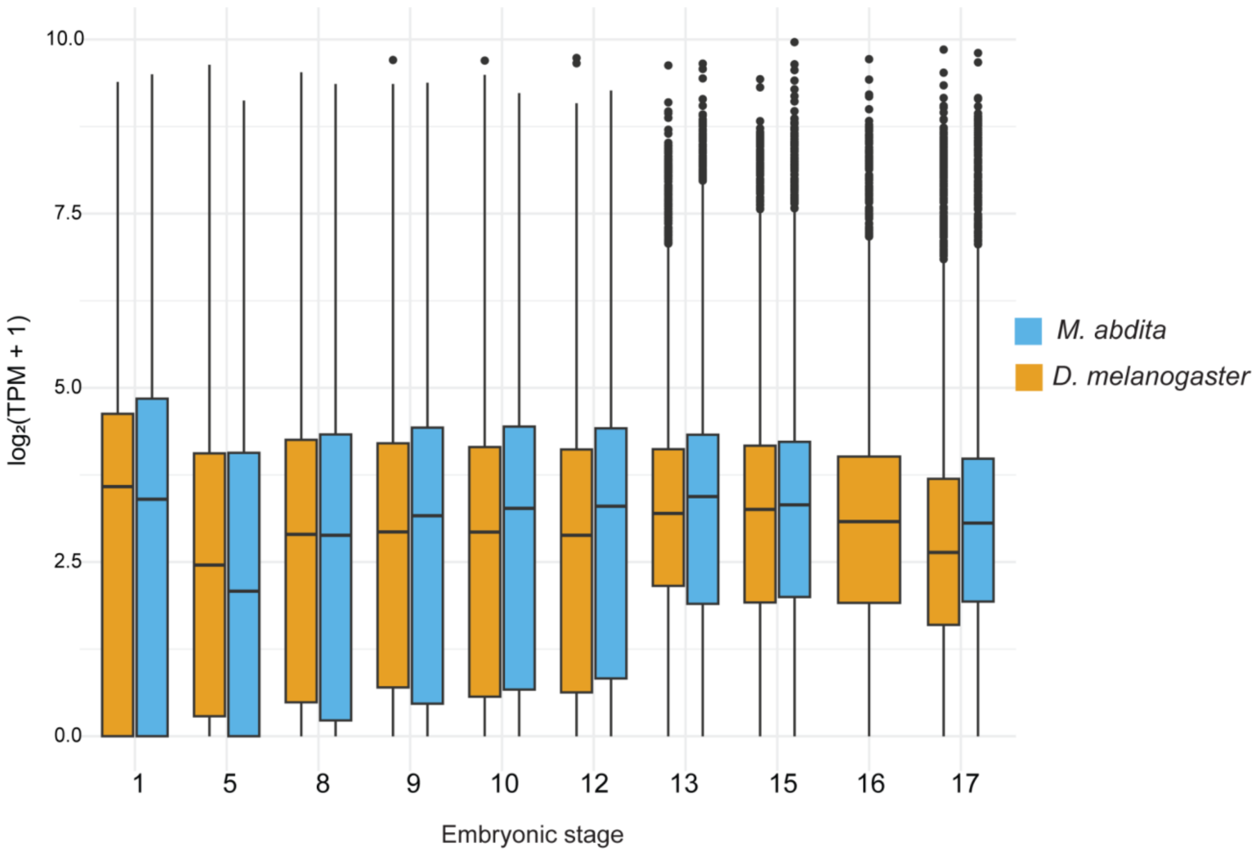

Fig. S8. Boxplot of gene expression in *D. melanogaster* (yellow) and *M. abdita* (blue) by embryonic stage. The y-axis represents log-transformed transcript abundance (TPM), and the x-axis represents the developmental stages. The boxes show the interquartile range (IQR), with the median indicated by the horizontal line. Black dots indicate outliers.

Development • Supplementary information

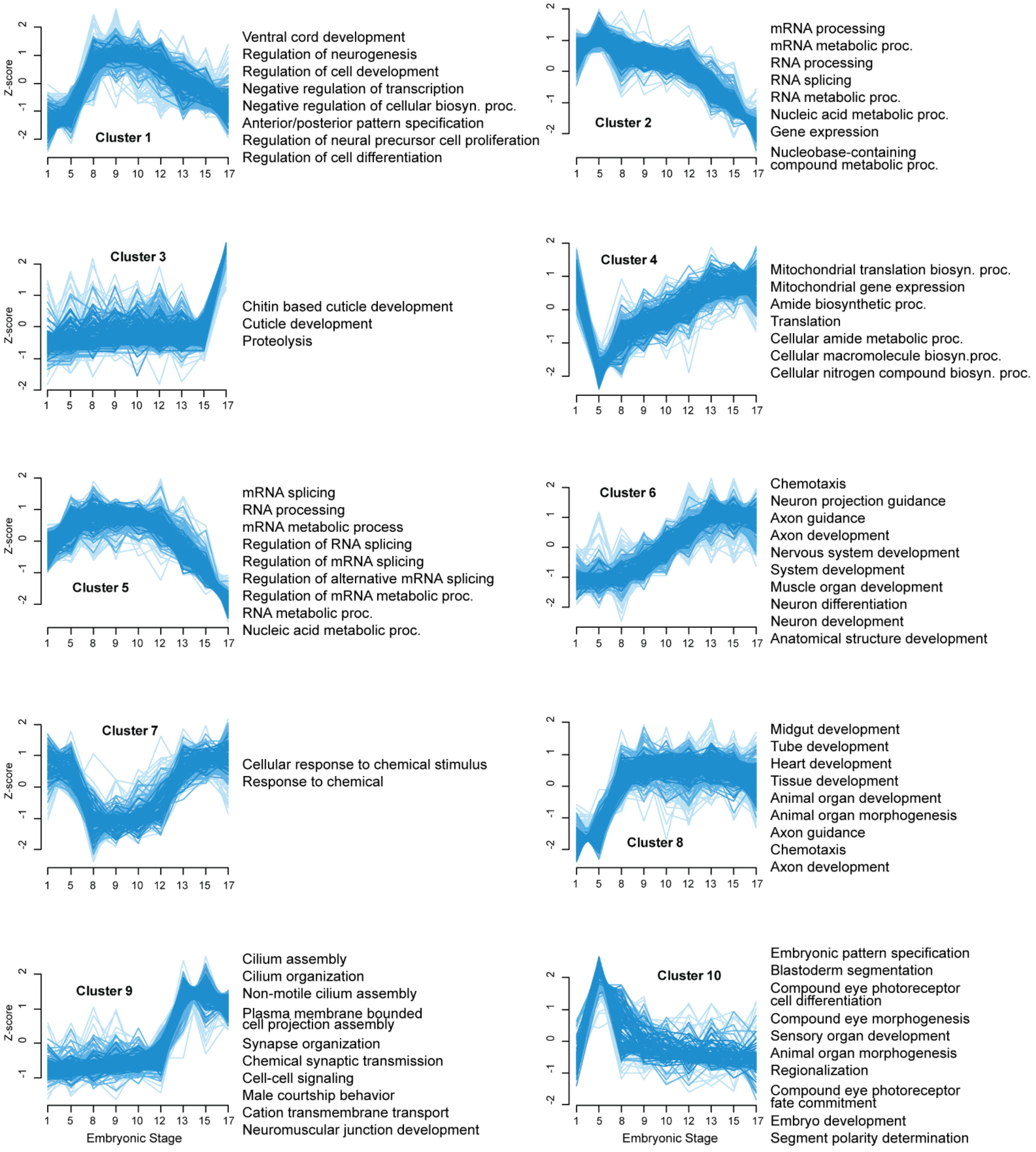

Development • Supplementary information

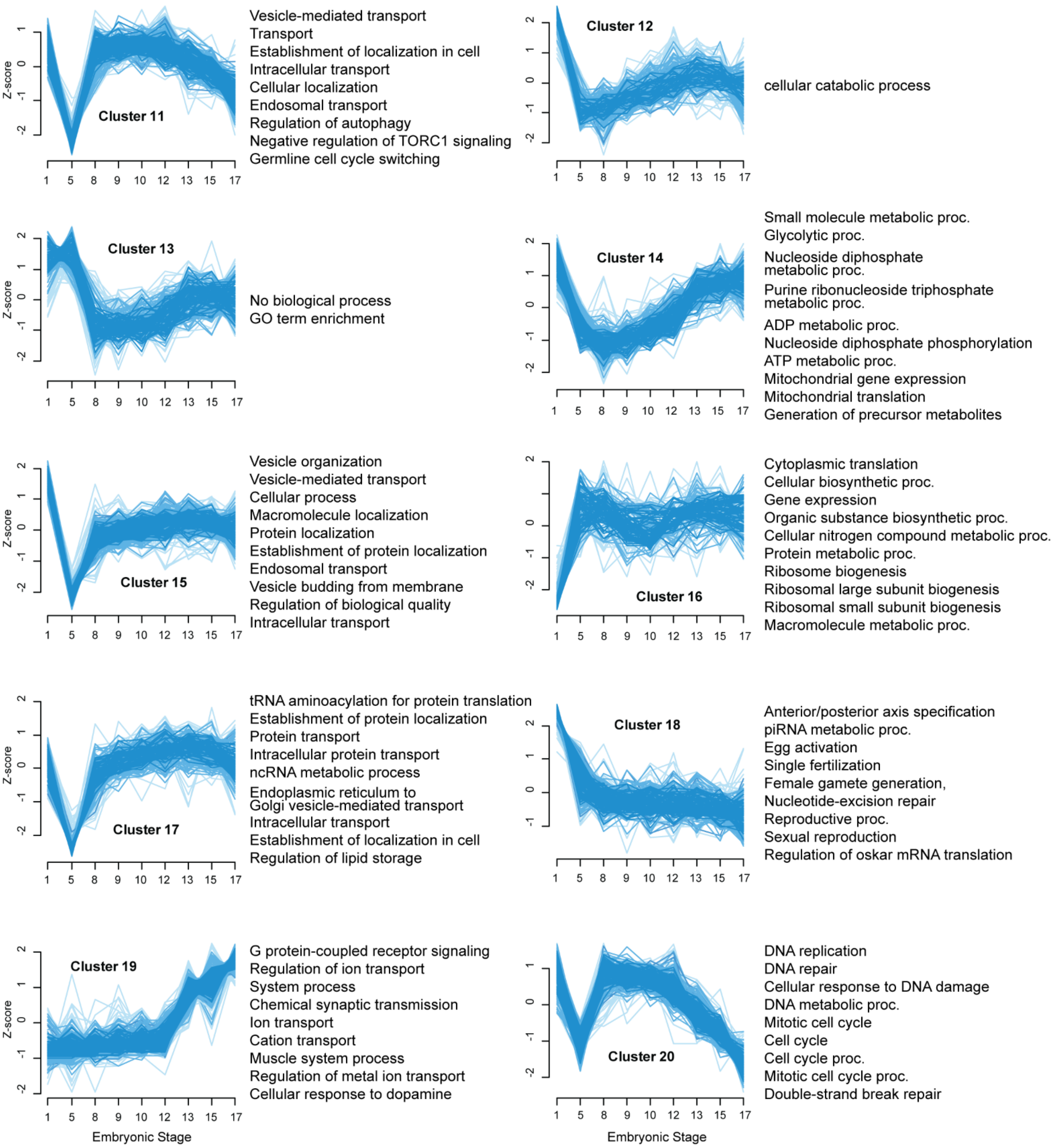

Development • Supplementary information

Development: doi:10.1242/dev.204732: Supplementary information

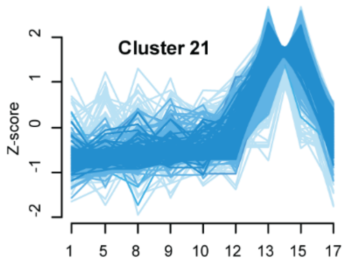

Cilium assembly
Microtubule-based movement
Plasma membrane bounded cell projection organization
Microtubule-based proc.
Cilium movement
Intraciliary transport
Cilium-dependent cell motility
Flagellated sperm motility
Microtubule-based transport

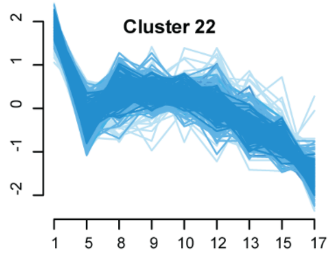

Macromolecule catabolic proc.
Protein catabolic proc.
Nucleic acid metabolic proc.
Nitrogen compound metabolic proc.
Cellular response to DNA damage
Ubiquitin-dependent protein catabolic proc.

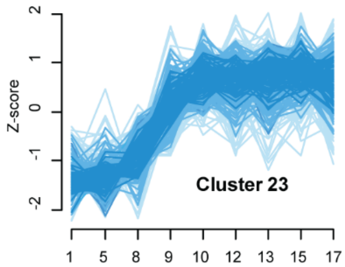

Respiratory system development
Open tracheal system development
Post-embryonic development
Chemotaxis
Taxis
Tube morphogenesis
Regulation of response to biotic stimulus
Positive regulation of defense response
Regulation of secondary metabolic proc.

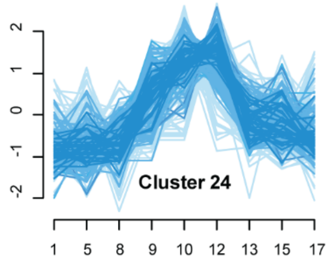

Ribosomal large subunit biogenesis
Maturation of LSU-rRNA
rRNA processing
ncRNA processing
RNA processing
RNA metabolic process
Maturation of LSU-rRNA
Organic cyclic compound metabolic pr
Nucleobase-containing compound metabolic proc.

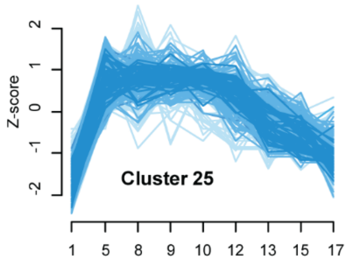

Digestive tract morphogenesis
Digestive tract development
Cell fate specification
Malpighian tubule development
Ectoderm and mesoderm interaction
Cell fate commitment
Hindgut morphogenesis
Tube morphogenesis
Tube development

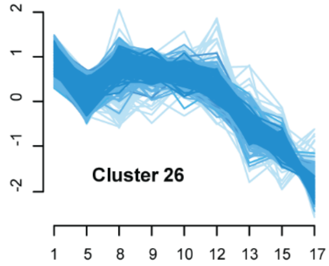

Mitotic cell cycle proc.
Mitotic cell cycle
Cell cycle proc.
Cell cycle
Nuclear chromosome segregation
Mitotic sister chromatid segregation
Chromosome segregation
Nuclear division
Regulation of cell cycle

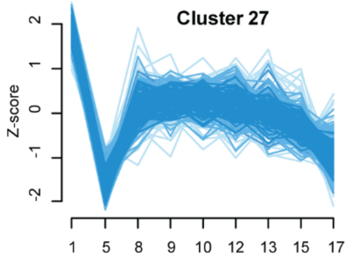

Proteasome-mediated ubiquitin-dependent protein catabolic proc.
Proteolysis involved in proteincatabolic proc.
Ubiquitin-dependent protein catabolic proc.
Macromolecule catabolic proc.
Protein ubiquitination
Cellular macromolecule metabolic proc.
Protein modification proc.

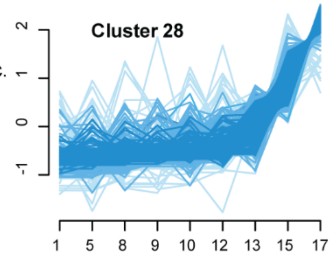

Chitin binding
Monooxygenase activity

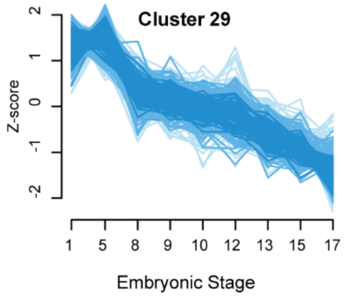

Regulation of catabolic proc.
Ubiquitin-dependent protein catabolic proc.
Cellular macromolecule catabolic proc.
Germ cell development
Regulation of proteasomal protein catabolic proc.
Protein modification by small protein conjugation or removal

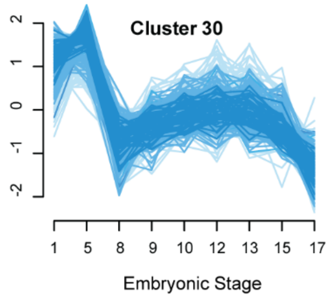

No BP GO terms enriched

Development • Supplementary information

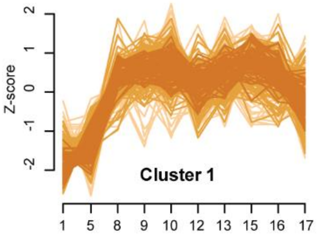

Chemotaxis
Axon guidance
Axon development
Neurogenesis
Generation of neurons
Nervous system development
Animal organ development
System development
Animal organ morphogenesis

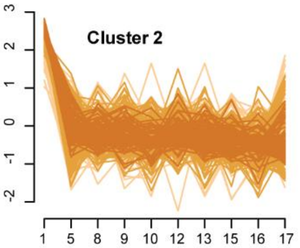

Anterior/posterior axis specification
Axis specification
Embryonic pattern specification
Oogenesis
Blastoderm segmentation
Female gamete generation
Maternal determination of anterior/posterior axis
Pole plasm assembly
Oocyte development

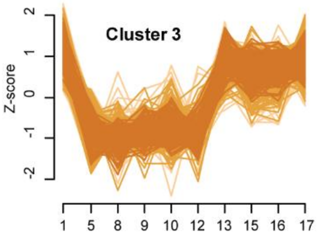

Monocarboxylic acid metabolic proc.
Glycolytic proc.
ATP metabolic proc.
Pyruvate metabolic proc.
Carbohydrate homeostasis
Glucose homeostasis
Nucleotide metabolic proc.
Carboxylic acid metabolic proc.
Small molecule metabolic proc.

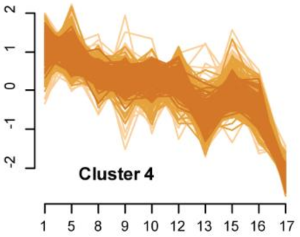

RNA processing
RNA metabolic proc.
Nucleic acid metabolic proc.
Histone modification
Regulation of gene expression
Regulation of metabolic proc.
Regulation of cellular metabolic proc.
Regulation of nitrogen compound metabolic proc.
Regulation of primary metabolic proc.
Regulation of cellular metabolic proc.

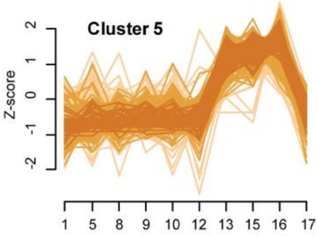

No BP GO terms

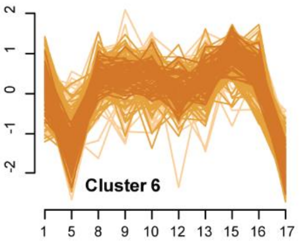

Retrograde vesicle-mediated transport
Ubiquitin-dependent ERAD pathway
Cellular proc.
Protein transport
Protein localization
Golgi vesicle transport
intra-Golgi vesicle-mediated transport
Endoplasmic reticulum to Golgi vesicle
Vesicle-mediated transport
Golgi organization

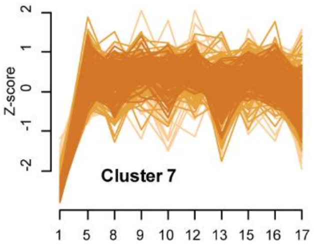

Cytoplasmic translation
Translation
Peptide metabolic proc
Cellular macromolecule biosyn. proc.
Nitrogen compound biosynthetic proc.
Gland development
Formation of primary germ layer
Tissue morphogenesis
Animal organ morphogenesis

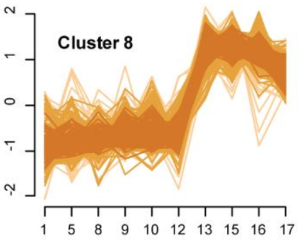

Neuron development
Neuron differentiation
Generation of neurons
Neurogenesis
Axon development
Nervous system development
System development
Plasma membrane bounded cell projection
Synaptic target recognition
Neuron projection development

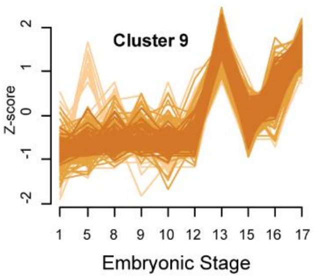

G protein-coupled receptor signaling
Signaling
Neuropeptide signaling
Metal ion transport
Cation transport
G protein-coupled receptor signaling
Chemical synaptic transmission
Sodium ion transport
System proc.

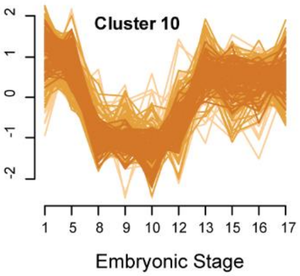

Cellular metabolic proc.

Development • Supplementary information

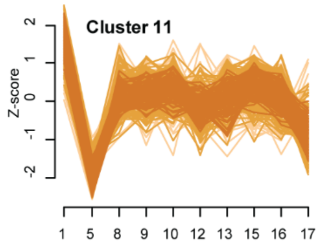

Protein transport
Nitrogen compound transport
Cellular macromolecule localization
Intracellular transport
Establishment of localization in cell
tRNA metabolic proc.
Cellular macromolecule metabolic proc.
Intracellular protein transport
Vesicle-mediated transport
tRNA aminoacylation for protein trans.

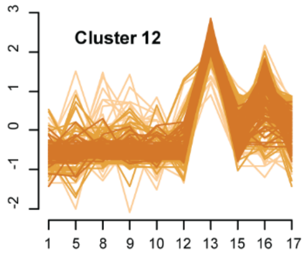

Chitin-based cuticle development
Body morphogenesis

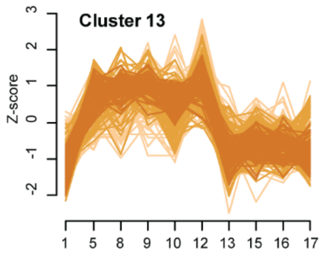

Cell fate specification
Ecdysone biosynthetic proc.
Cellular ketone metabolic proc.
Gastrulation
Organic hydroxy compound biosyn. proc.
Gastrulation involving germ band ext.
Embryonic morphogenesis
Cell fate commitment
Regulation of Notch signaling
Digestive tract development

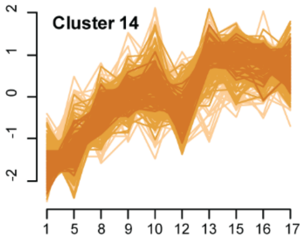

Regulation of tube architecture
Open tracheal system development
Central nervous system development
Cell adhesion
Animal organ development
Imaginal disc morphogenesis
Tube morphogenesis
Post-embryonic animal morphogenesis
System development
Tissue morphogenesis

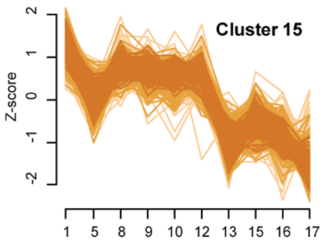

DNA replication
DNA metabolic proc.
DNA repair
Cellular response to DNA damage
DNA recombination
Double-strand break repair
Cell cycle proc.
Cell cycle
Mitotic cell cycle proc.

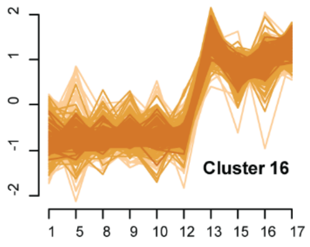

Modulation of chemical synaptic transmission
Synapse organization
Cell junction organization
Synaptic signaling
Chemical synaptic transmission
Cell-cell signaling
Regulation of membrane potential
System process
Signaling
Muscle system process

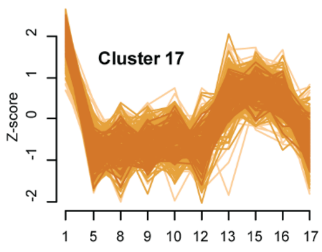

No BP GO term

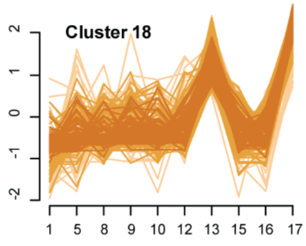

Chitin-based cuticle development
Cuticle development

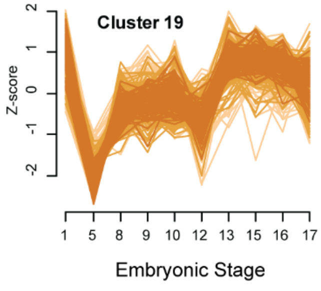

Protein targeting
Protein targeting to mitochondrion
Mitochondrial gene expression
Mitochondrion organization
Intracellular protein transport
Protein transport
Nitrogen compound transport
Mitochondrial RNA metabolic proc.
Protein insertion into mito. mem.

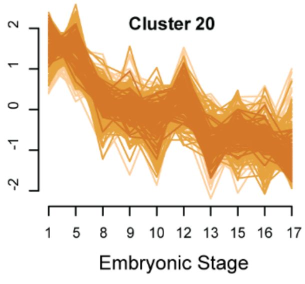

Dependent protein catabolic proc.
Meiotic nuclear division
Macromolecule catabolic proc.
Meiotic cell cycle process
Nuclear division
Homologous chromosome pairing at meiosis
Cellular catabolic process
Organic substance catabolic process
Synaptonemal complex organization

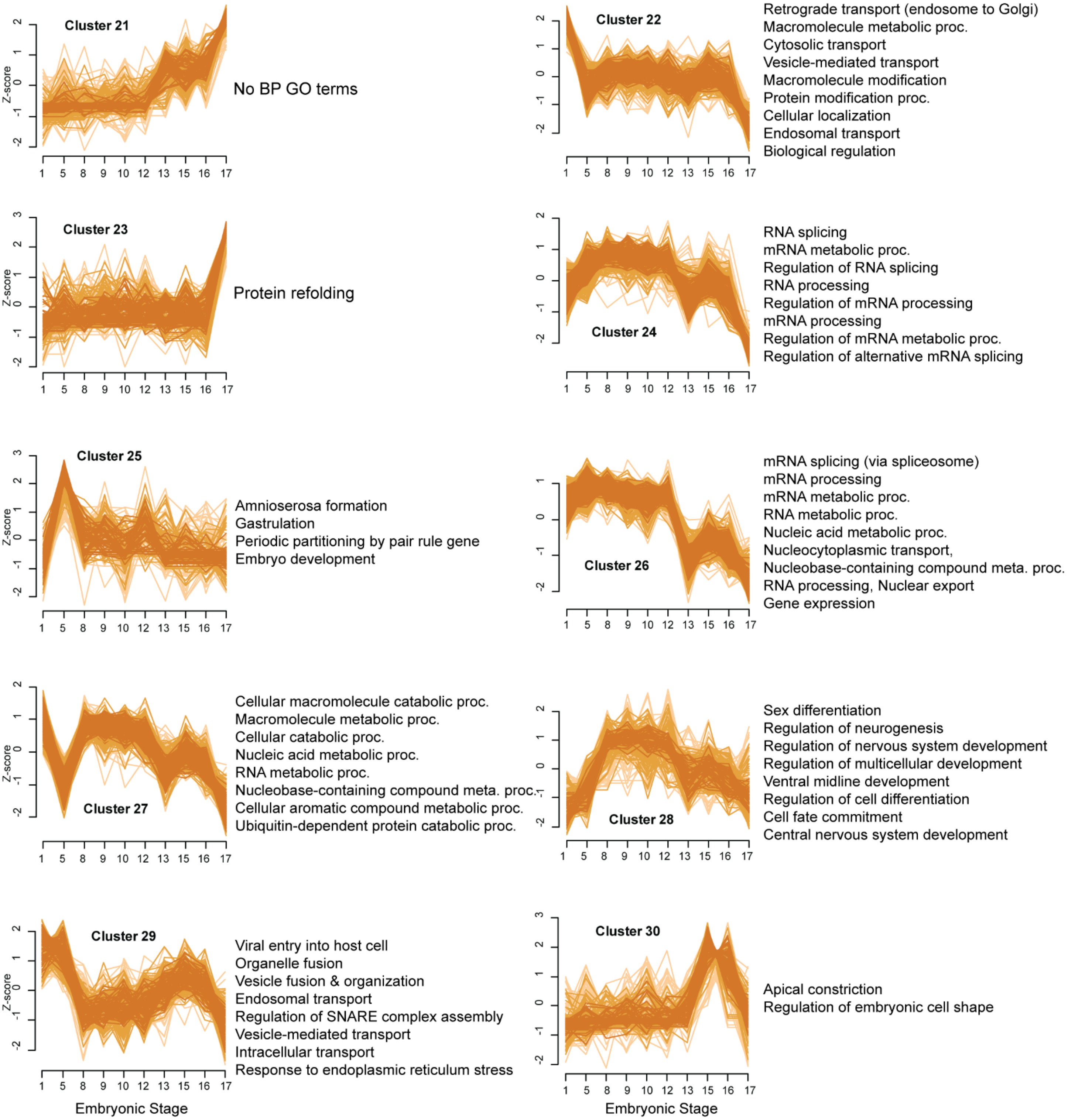

Development • Supplementary information

Fig. S9. Mfuzz gene cluster plots and enriched functional annotations during embryogenesis in *M. abdita* and *D. melanogaster*. Plots of Mfuzz gene clusters during embryogenesis (membership score > 0.7). *M. abdita* plotted in blue and *D. melanogaster* in orange. Each line is the z-score of a gene across embryonic stages. Z-score is equivalent to the expression of the gene at a specific stage minus the mean across all stages divided by the standard deviation across all stages. A z-score of +2 at a specific stage indicates that the gene is +2 standard deviations away from the mean. The Mfuzz generated cluster names (e.g., "Cluster 1") are arbitrary but retained for continuity. For both species, we specified 30 clusters. To the right of each plot, is that cluster's top 10 enriched Biological Process Gene Ontology (BP GO) terms.

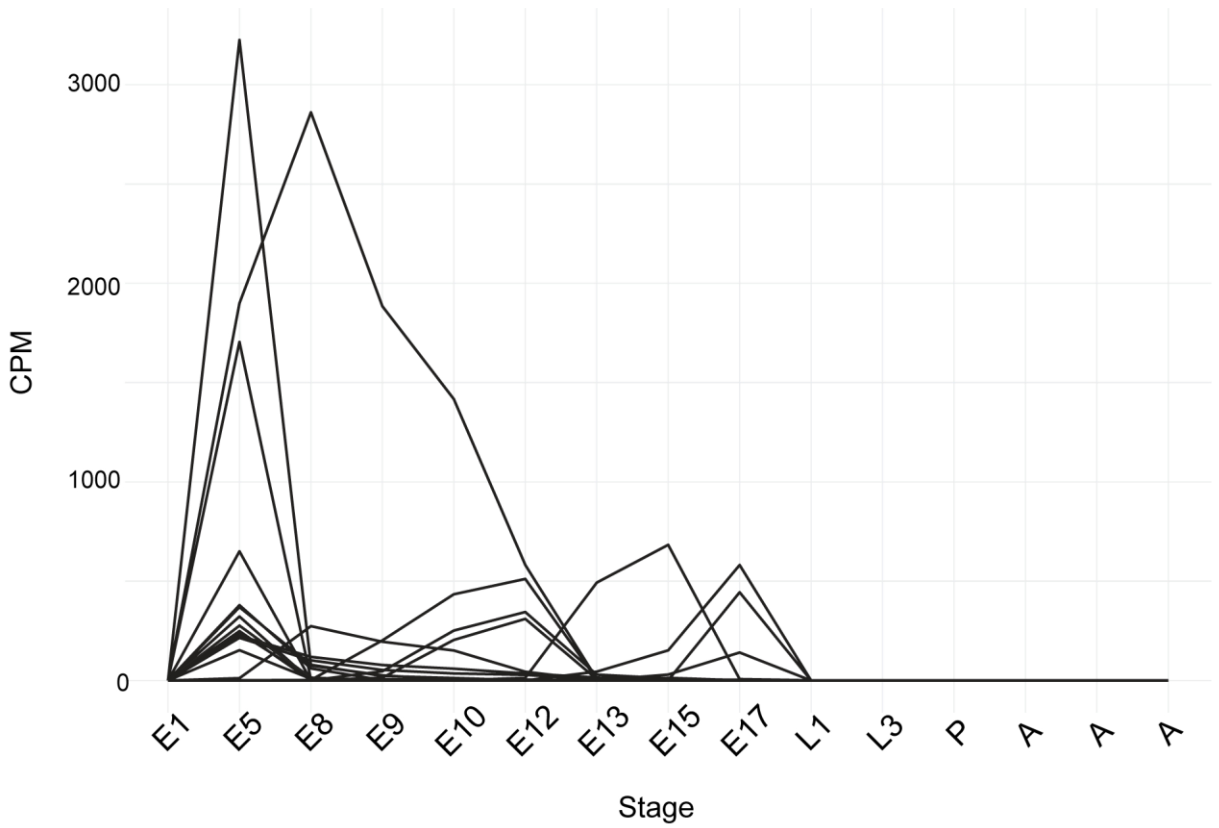

Fig. S10. Expression profiles of 24 *M. abdita* orphan genes with expression limited to embryonic stages. E indicates embryonic, L indicates larval, P indicates pupal, and A indicates adult (multiple samples). Expression is in counts per million (CPM).

Development • Supplementary information

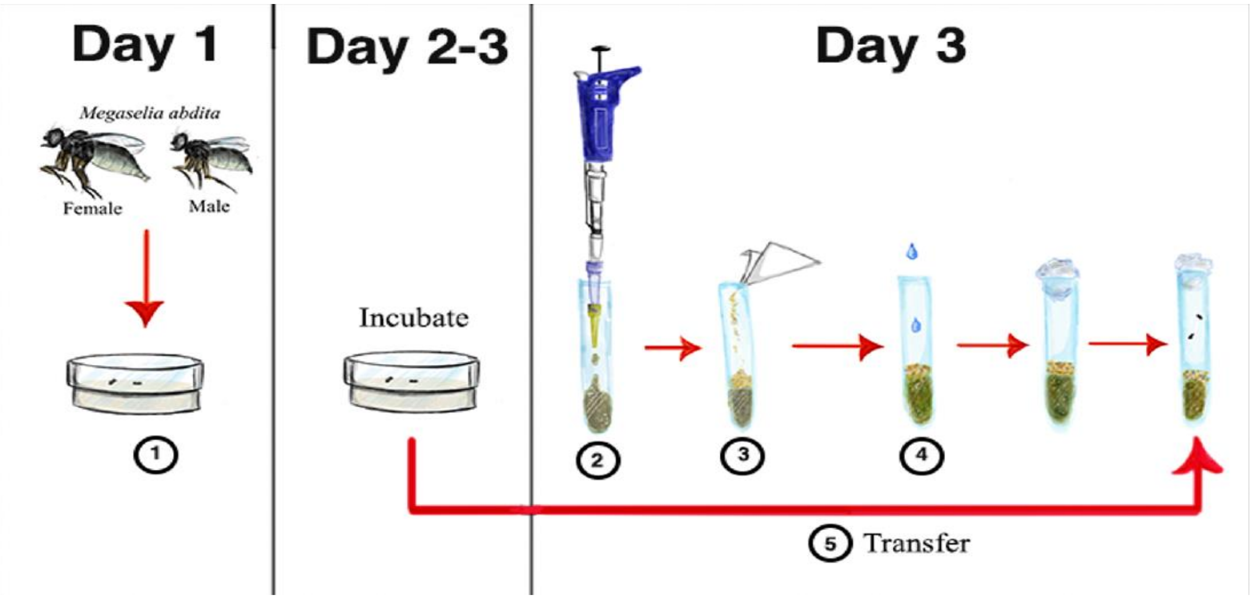

Fig. S11. General overview of the protocol for the single crosses of *M. abdita*. Step 1: Virgin flies are collected and incubated in 2% agar-water gel plates for 2 days with a single male. Step 2: Food vials are prepared the day of transfer. Step 3: Once the agar has solidified, 0.1g additional food is added using weighing paper. Step 4: 200ul of water is added. Step 5: Flies are transferred into the food vials which are plugged with rayon and then incubated for ~21 days.

Development • Supplementary information

Table S1. Scaffold lengths of *M. abdita's* genome assembly.

Scaffold	Length (bp)
1	222,469,999
2	212,802,314
3	155,577,902
4	1,302,958
5	455,293
6	55,021
7	35,625
8	29,721
9	17,979
10	17,687
11	17,313
12	13,866
13	13,720
14	10,567
15	4,999

Table S2. Comparison of *D. melanogaster* and *M. abdita's* protein-coding genes exon, transcript, and full gene length in base pairs (bp). All *D. melanogaster* non-protein coding genes were removed before analysis.

	Min	Median	Mean	Max
**Exons**				
D. melanogaster	1	250	553	28,074
M. abdita	3	293	505	19,709
**Transcript (all isoforms)**				
D. melanogaster	152	1,700	2,353	71,382
M. abdita	306	1,581	2,053	43,640
**Gene**				
D. melanogaster	162	2,159	6,960	1,965,856
M. abdita	306	6,650	19,479	476,305

Development • Supplementary information

Table S3. Strength, signal, and false discovery rates for each enriched GO term from main text figure 4. Strength of enrichment is (Log_{10}(observed/expected)), false discovery rate is p-value corrected for multiple testing with Benjamini-Hochberg, and signal is the weighted harmonic mean between observed/expected ratio and -log(FDR).

Cluster	Go Terms	Strength	Signal	FDR	Strength	Signal	FDR
Early embryonic patterning	Anterior/posterior axis specification,embryo	1.04	0.91	0.00017	1.09	1.34	1.02E-06
	Female gamete generation	0.66	0.81	2.40E-05	0.69	0.98	1.02E-06
	germ cell development	0.58	0.67	0.00017	0.65	0.94	1.02E-06
	pole plasm assembly	1.07	0.52	0.0121	1.21	0.99	0.00014
	piRNA metabolic process	1.45	0.87	0.00074	1.35	0.59	0.0077
	cell differentiation	0.35	0.38	0.0086	0.39	0.51	0.00067
	RNA localization	0.79	0.44	0.0175	0.79	0.41	0.0245
System Development	animal organ development	0.48	0.57	0.0003	0.64	0.93	6.14E-08
	anatomical structure development	0.31	0.4	0.0026	0.4	0.58	7.32E-06
	regulation of neurogenesis	0.95	0.73	0.001	1.14	1.32	2.31E-06
	sensory organ development	0.55	0.34	0.0348	0.78	0.93	7.32E-06
	ventral cord development	1.15	0.89	0.00034	1.18	0.9	0.00041
	regionalization	0.61	0.51	0.0036	0.67	0.62	0.00079
	cell fate commitment	0.73	0.61	0.0016	0.93	1.16	1.24E-06
	regulation of cell population proliferation	0.73	0.42	0.0192	0.77	0.5	0.0092
	regulation of RNA metabolic process	0.48	0.58	0.00027	0.53	0.68	1.29E-05
	regulation of metabolic process	0.34	0.46	0.00066	0.37	0.48	0.00028
Signaling	nervous system process	0.4	0.56	0.00064	0.45	0.61	0.00032
	neuropeptide signaling pathway	0.76	0.53	0.0052	1.01	1.15	7.77E-06
	G protein-couple receptor signaling	0.71	1.17	1.65E-07	0.86	1.76	1.42E-11
	metal ion transport	0.46	0.38	0.0176	0.69	0.94	7.77E-06
	cell communication	0.28	0.52	0.00044	0.36	0.66	1.94E-05
	chemical synaptic transmission	0.6	0.7	0.00027	0.67	0.77	0.00014
	ion transport	0.4	0.59	0.00032	0.45	0.61	0.00027
	synapse organization	0.59	0.59	0.0013	0.64	0.56	0.0026
	regulation of membrane potential	0.69	0.5	0.0068	0.79	0.58	0.0032
	transmembrane transport	0.35	0.55	0.00041	0.35	0.44	0.0042
	cellular response to dopamine	1.14	0.73	0.0012	1.13	0.47	0.0156
Cuticle Development	Chitin-based cuticle development	0.9	1.72	8.23E-11	1.06	1.71	2.18E-09
DNA replication, repair, and cell cycle regulation	DNA-templated DNA replication	1.24	2.77	1.22E-14	1.2	3.84	5.15E-24
	DNA repair	1.06	2.62	7.44E-17	1.03	3.51	7.07E-29
	double-strand break repair	1.06	1.72	3.25E-09	1.09	3.09	6.88E-20
	DNA metabolic process	0.96	2.57	5.30E-20	0.98	3.73	1.41E-40
	cell cyle	0.85	2.46	2.98E-25	0.82	2.91	1.68E-41
	mitotic cell cycle	0.92	2.52	4.76E-21	0.86	2.83	8.01E-31
	DNA recombination	1.1	1.86	9.41E-10	1.11	3.05	5.54E-19
	nuclear division	0.85	1.5	1.45E-09	0.89	2.56	1.44E-21
	nuclear chromosome segregation	0.88	1.18	1.29E-06	0.98	2.61	2.87E-18
	cellular response to DNA damage	0.99	2.38	4.22E-16	1	3.49	3.03E-31

Table S4. Coding and protein sequence characteristics of orphan genes in *M. abdita*. The table includes coding sequence (nucleotide) and amino acid sequence (protein) characteristics for each orphan gene (Gene ID as it appears in the genome annotation). Genes highlighted in blue have an amino acid sequence with a stability index classifying them as "stable." "n.s." indicates "no significant match" in sequence similarity searches using NCBI’s blast.

	Nucleotide					Protein				
Gene ID	ORF length (AA)	Fickett Score	Isoelectric Point	Coding Probability	nblast	alphafold (pTM)	Instability Index	Aliphatic Index	Average of hydropathicity	pblast
evm.TU.Scaffold_3_155577901.980	571	0.41	6.46	1.00	n.s.	0.57	38.92	99.51	-0.12	n.s.
evm.TU.Scaffold_1_222469999.1571	174	0.39	6.09	0.98	n.s.	0.53	36.79	85.66	-0.44	n.s.
evm.TU.Scaffold_1_222469999.2459	102	0.46	8.08	0.53	n.s.	0.39	30.12	115.74	0.60	n.s.
evm.TU.Scaffold_2_212802314.799	139	0.45	5.06	0.97	n.s.	0.36	37.03	77.75	-0.03	n.s.
evm.TU.Scaffold_1_222469999.1775	105	0.44	4.54	0.79	n.s.	0.32	28.32	139.71	0.82	n.s.
evm.TU.Scaffold_1_222469999.6	264	0.43	9.66	1.00	n.s.	0.29	29.60	76.84	-0.15	n.s.
evm.TU.Scaffold_2_212802314.3488	181	0.44	4.32	1.00	n.s.	0.21	19.35	88.89	-0.04	n.s.
evm.TU.Scaffold_3_55577901.555	459	0.36	5.30	1.00	n.s.	0.19	39.71	64.02	-0.73	n.s.
evm.TU.Scaffold_1_22469999.3564	270	0.43	9.08	1.00	n.s.	0.16	36.66	82.90	-0.23	n.s.
evm.TU.Scaffold_1_222469999.1521	281	0.42	9.57	1.00	n.s.	0.12	52.75	59.14	-0.78	n.s.
evm.TU.Scaffold_1_222469999.394	229	0.44	4.71	1.00	n.s.	0.34	48.89	108.25	-0.07	n.s.
evm.TU.Scaffold_1_222469999.1484	182	0.42	4.32	1.00	n.s.	0.30	87.99	72.76	-1.02	n.s.
evm.TU.Scaffold_1_222469999.4787	212	0.38	9.03	0.99	n.s.	0.18	63.17	69.29	-1.15	n.s.
evm.TU.Scaffold_3_155577901.2852	193	0.47	5.30	1.00	n.s.	0.21	56.40	62.97	-0.80	n.s.
evm.TU.Scaffold_1_222469999.5029	1048	0.41	5.12	1.00	n.s.	0.19	60.36	75.19	-0.58	n.s.
evm.TU.Scaffold_1_222469999.1143	207	0.43	4.22	1.00	n.s.	0.22	55.67	97.95	-0.47	n.s.
evm.TU.Scaffold_3_55577901.1386	217	0.34	6.12	0.99	n.s.	0.31	49.03	81.25	-0.63	n.s.
evm.TU.Scaffold_3_155577901.2294	369	0.46	5.06	1.00	n.s.	0.25	64.49	54.81	-1.21	n.s.
evm.TU.Scaffold_1_222469999.1464	461	0.41	6.05	1.00	n.s.	0.24	44.23	87.04	-0.68	n.s.
evm.TU.Scaffold_1_222469999.231	100	0.45	5.61	0.59	n.s.	0.36	46.90	107.37	-0.35	n.s.
evm.TU.Scaffold_1_222469999.29	245	0.43	4.68	1.00	n.s.	0.26	53.24	74.30	-0.80	n.s.
evm.TU.Scaffold_1_222469999.3878	331	0.45	7.53	1.00	n.s.	0.26	43.83	70.79	-0.56	n.s.
evm.TU.Scaffold_2_212802314.823	274	0.44	8.69	1.00	n.s.	0.18	55.28	64.98	-1.07	n.s.
evm.TU.Scaffold_3_55577901.923	217	0.43	4.40	1.00	n.s.	0.18	50.50	92.04	-0.44	n.s.

Development • Supplementary information

Table S5. All RNA-Seq sample names, accession numbers, metadata (from our study and NCBI).

Sample Name	Developmental Stage	Source	BioSample	BioProject	SRA Run
M_abdita_F_RNA	Adult	Mahajan and Bachtrog. 2017.	SAMN06909123	PRJNA385725	SRR5559325
M_abdita_M_RNA	Adult	Mahajan and Bachtrog. 2017.	SAMN06909124	PRJNA385725	SRR5559340
RINSinITBGRAAPEI-126	Adult	Pauli et al. 2018.	SAMN03223122	PRJNA267960	SRR1695360
mega-hiseq1	Pooled Embryos (late blastoderm though early germband extension)	Jiménez-Guri et al. 2013.	SAMEA1572180	PRJEB3172	ERR196167
Megaselia_454_ok	Pooled Embryos (late blastoderm though early germband extension)	Jiménez-Guri et al. 2013.	SAMEA1572181	PRJEB3172	ERR194165
Mab_Stage_13	Embryo - Stage 13	This study	SAMN45895270	PRJNA1200075	SRR31763336
Mab_Stage_15	Embryo - Stage 15	This study	SAMN45895271	PRJNA1200075	SRR31763335
Mab_Stage_17	Embryo - Stage 17	This study	SAMN45895272	PRJNA1200075	SRR31763327
Mab_Stage_1	Embryo - Stage 1	This study	SAMN45895273	PRJNA1200075	SRR31763321
Mab_Stage_5	Embryo - Stage 5	This study	SAMN45895274	PRJNA1200075	SRR31763320
Mab_Stage_8	Embryo - Stage 8	This study	SAMN45895275	PRJNA1200075	SRR31763318
Mab_Stage_9	Embryo - Stage 9	This study	SAMN45895276	PRJNA1200075	SRR31763317
Mab_Stage_10	Embryo - Stage 10	This study	SAMN45895277	PRJNA1200075	SRR31763316
Mab_Stage_12	Embryo - Stage 12	This study	SAMN45895278	PRJNA1200075	SRR31763319
Mab_L1	Larva - 1st instar	This study	SAMN45895279	PRJNA1200075	SRR31763315
Mab_L3	Larva - 3rd instar	This study	SAMN45895280	PRJNA1200075	SRR31763334
Mab_pupa	Pupa - day 1	This study	SAMN45895281	PRJNA1200075	SRR31763333
Dmel_Stage_13	Embryo - Stage 13	This study	SAMN45895282	PRJNA1200075	SRR31763332
Dmel_Stage_15	Embryo - Stage 15	This study	SAMN45895283	PRJNA1200075	SRR31763331
Dmel_Stage_16	Embryo - Stage 16	This study	SAMN45895284	PRJNA1200075	SRR31763330
Dmel_Stage_17	Embryo - Stage 17	This study	SAMN45895285	PRJNA1200075	SRR31763329
Dmel_Stage_1	Embryo - Stage 1	This study	SAMN45895286	PRJNA1200075	SRR31763328
Dmel_Stage_5	Embryo - Stage 5	This study	SAMN45895287	PRJNA1200075	SRR31763326
Dmel_Stage_8	Embryo - Stage 8	This study	SAMN45895288	PRJNA1200075	SRR31763325
Dmel_Stage_9	Embryo - Stage 9	This study	SAMN45895289	PRJNA1200075	SRR31763324
Dmel_Stage_10	Embryo - Stage 10	This study	SAMN45895290	PRJNA1200075	SRR31763323
Dmel_Stage_12	Embryo - Stage 12	This study	SAMN45895291	PRJNA1200075	SRR31763322

Table S6. Fluorescent *in situ* hybridization chain reaction probe sequences.

Available for download at
https://journals.biologists.com/dev/article-lookup/doi/10.1242/dev.204732#supplementary-data
